# Supplementary material for: The Preparation and Study of Ethylene Glycol-Modified Graphene Oxide Membranes for Water Purification
Source: Polymers (Basel). 2019 Jan 22;11(2):188. doi: 10.3390/polym11020188 (PMC6418752; doi:10.3390/polym11020188)
Supplement: Supplementary file 1 [file polymers-11-00188-s001.pdf]

Supplementary materials

Experimental details

Graphene oxide nanosheets were synthesized from natural graphite powder by the modified Hummers' method. 3g of graphite was added gradually into 120mL H_2SO_4 (98.0%), and kept below 283K. Then, 15g of $KMnO_4$ (5 times apart) was added into the mixture under stirring and cooling. After mixing evenly, further stirred at 308K for 2 hours, DI water was added, and 30% H_2O_2 was added and the color of the mixture changed to bright yellow. The product was washed with hydrochloric acid and DI water until neutral.

Figure

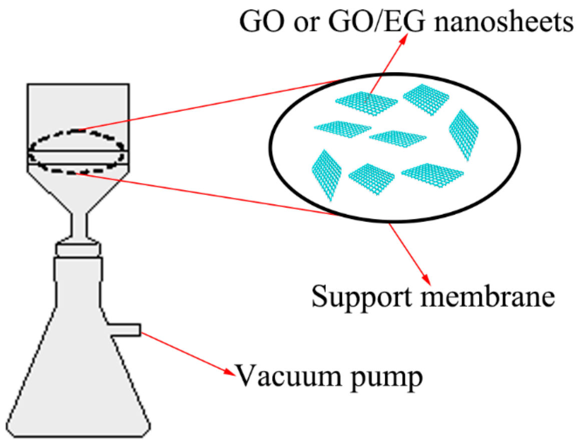

Fig. S1 schematic diagram of vacuum membrane forming device.

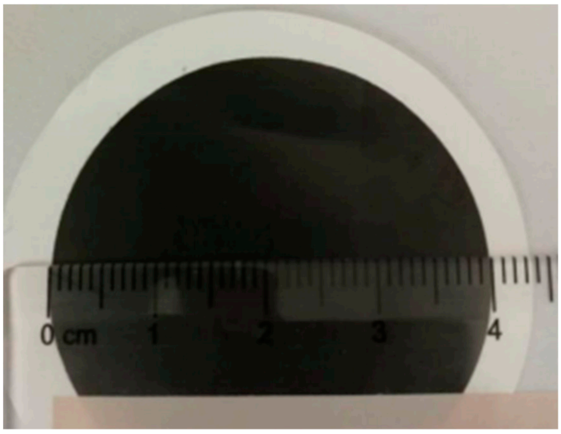

Fig. S2 the effective diameter of membranes.

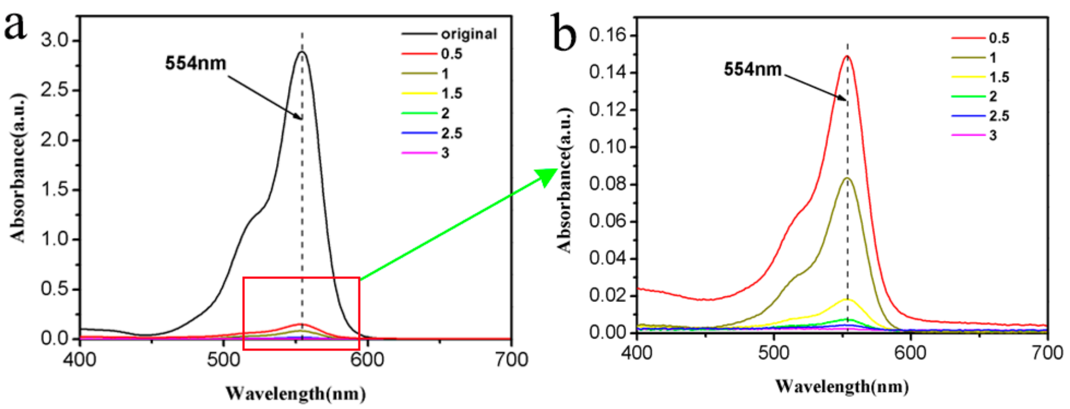

Fig. S3 the absorbance spectra correspsonding to RhB of GO/EG membrane.

As shown in the Fig. S3, we can observe that the absorption wavelength of RhB is 554 nm. As shown in the Fig. S3 (a), we can observe that the absorbance of the original RhB solution is much higher than the absorbance of the filtrate (0.5, 1, 1.5, 2, 2.5 and 3 represent the RhB filtrate of the corresponding GO/EG membrane, respectively.). We can obtain that GO/EG membranes have a significant rejection effect of RhB. As shown in the Fig. S3 (b), we can find a positive correlation between the rejection and the thickness of the GO/EG membrane.

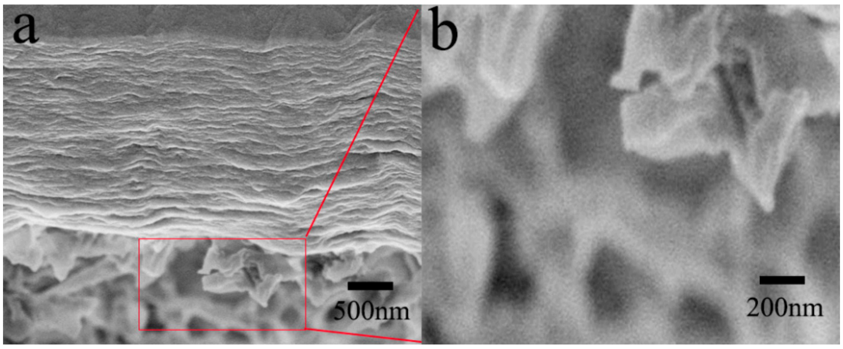

Fig. S4 (b) shows the cross-section of the support membrane.

As shown in the Fig. S4 (b), we can observe that the internal structure of the support membrane is very large, and water molecules can easily pass through the support membrane, which has a negligible effect on the performance of the GO/EG membrane. This can also be proved in the Table S1, below.

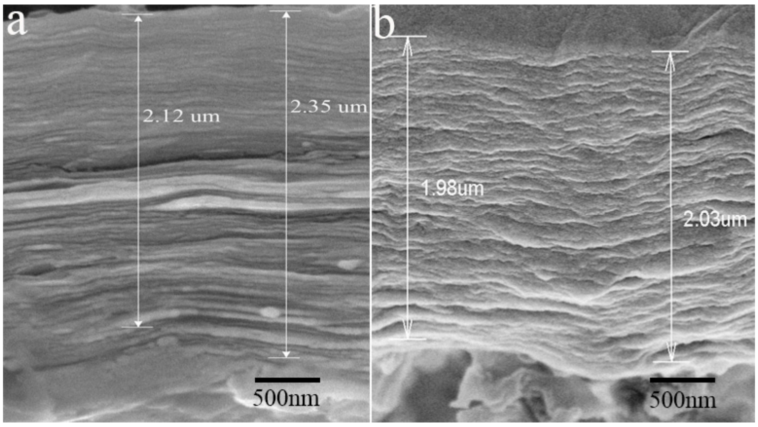

Fig. S5 the thickness of GO (a) and GO/EG (b) membranes

The thickness of GO and GO/EG membranes can be measured directly in the FESEM image, as shown in the Fig. S4. The thickness (dry state) of the membranes is about 2 um.

Table

Table S1

The water permeability comparison of support membrane, GO membrane and GO/EG membrane

Membrane	Support Membrane	GO Membrane	GO/EG Membrane
**Volume of water(10^{-3}L)**	10	10	10
**Permeability($L\ m^{-2}\ h^{-1}\ bar^{-1}$)**	$3\times10^{4}\pm1\times10^{3}$	205 ±10 (Max)	110 ±5 (Max)
